# Homogeneous Crystal Nucleation of Poly(3-hydroxybutyrate): Kinetics, Stability, and Cluster-Size Distribution

**DOI:** 10.1021/acs.macromol.5c02381

**Published:** 2025-12-18

**Authors:** Katalee Jariyavidyanont, Rui Zhang, Christoph Schick, René Androsch

**Affiliations:** † Interdisciplinary Center for Transfer-oriented Research in Natural Sciences (IWE TFN), 9176 Martin Luther University Halle-Wittenberg, Halle/Saale 06099, Germany; § Institute of Physics and Competence Centre CALOR, 9187 University of Rostock, Rostock 18051, Germany

## Abstract

The kinetics of homogeneous crystal nucleation in poly­(3-hydroxybutyrate) (P3HB) near its glass transition temperature (*T*_g_) was investigated using fast scanning calorimetry (FSC) combined with Tammann’s two-stage crystal nuclei development method. Crystallization on cooling is fully suppressed at rates of a few K/s, while the formation of crystal nuclei is inhibited only at higher rates, above around 100 K/s. The formation of homogeneous nuclei is fastest at around 40 °C (approximately 30 K above *T*_g_), with an onset time of their formation of about 1 s, which is two orders of magnitude shorter than the crystallization onset time. To evaluate their thermal stability, a modified Tammann nucleation experiment involving a temperature spike before the growth stage was employed. The number of homogeneous nuclei of supercritical size at the growth temperature formed under the given conditions remained nearly constant upon heating up to about 100 K above their formation temperature. Beyond this limit, the number of surviving nuclei drastically decreased, and complete dissolution was observed at about 120 K above the temperature of their formation. Besides the finding of an exceptionally high stability of homogeneous crystal nuclei of P3HB, the sharp disappearance of the number of nuclei within a narrow temperature range of 10–15 K suggests a sharp truncation of the nuclei-size distribution for larger nuclei.

## Graphical abstract

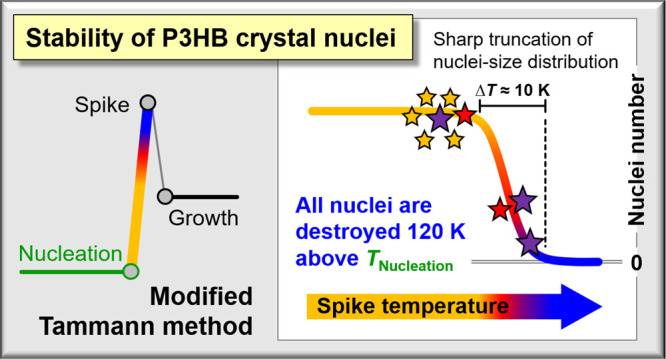

## 1 Introduction

Poly­(3-hydroxybutyrate) (P3HB) is a member of the poly­(hydroxyalkanoate) (PHA) family. It is a biobased and biodegradable aliphatic polyester, commonly synthesized via bacterial fermentation.^1,2^ This process uses bacteria, such as *Cupriavidus necator*, *Azohydromonas lata*, and recombinant *Escherichia coli*, to convert carbon or energy sources (like glucose, starch, molasses, or vegetable oils) into P3HB granules inside their cells.^1–3^ The granules serve as a carbon and energy reserve that the bacteria can later metabolize when external nutrients become scarce. It shows biodegradable behavior in all aerobic and anaerobic environments.^4–6^ This enables the development of products that are completely compostable and soil- and marine-biodegradable, offering an alternative solution to the environmental problems of plastic pollution and landfill accumulation.^5,6^ In addition, it is non-toxic to human cells and tissues. It can degrade through hydrolytic and enzymatic pathways, ensuring a gradual resorption without harmful byproducts or inflammation.^7–9^ Due to its excellent biodegradability and biocompatibility, it is used in diverse applications across food packaging as an alternative to polypropylene (PP),^10–12^ in agricultural applications such as mulching films and a coating for controlled-release fertilizers,^13,14^ and in various medical applications like surgical materials, tissue engineering scaffolds, and drug delivery systems.^9,15,16^

P3HB is a semicrystalline polymer in which the chain-ordering process or crystallization takes place at reasonable rates between its glass transition temperature (*T*_g_) of approximately 0 °C and equilibrium melting temperature (*T*_m,0_) of 195 °C.^17–19^ It should be noted that these *T*_g_ and *T*_m,0_ values are characteristic of P3HB with a molar mass in the range of a few hundred thousand to a few million g/mol.^20,21^ It exhibits inherently high crystallinity (typically over 60%) due to its high stereoregularity,^22–24^ which promotes efficient chain packing during primary crystallization. Furthermore, since its *T*_g_ is below ambient temperature, secondary crystallization processes contribute to a progressive increase in crystallinity during storage or annealing, even at room temperature.^25,26^ Numerous works provide information about the kinetics of melt-crystallization of bacterial P3HB and its structure formation at low supercooling of the melt, quantified by conventional differential scanning calorimetry (DSC) that typically offers scanning rates up to a few hundred K/min.^27^ Upon cooling at rates ranging from 40 to 2.5 K/min, crystallization of P3HB occurs in the temperature range of 60 to 110 °C, yielding crystallinity in the range of 20–60%, respectively.^28–30^ Complete vitrification of the amorphous glass is obtained when the equilibrium melt is quenched at rates above 200 K/min to below *T*_g_.^30–33^ While some reports suggest a cooling rate higher than 500 K/min, this discrepancy might be attributed to variations in the material composition, such as the presence of additives or nucleating agents.^34^ Subsequent heating of the amorphous glass at rates of a few ten K/min reveals an exothermic cold-crystallization peak at 50–60 °C in DSC scans.^29–33^ As such, cooling at rates of a few hundred K/min is sufficient to suppress crystallization (crystal growth). However, it may not be fast enough to prevent the formation of homogeneous crystal nuclei. Even if the cooling rate is sufficient to prevent nuclei formation, those may also form during aging the glass, or on subsequent heating of the system. These nuclei then can grow to crystals if the temperature is increased into a temperature range where the growth rate is high.

The kinetics of isothermal melt-crystallization at specific temperatures has been investigated, suggesting a monomodal temperature-distribution of crystallization half-times with a minimum of several ten seconds at 60–70 °C.^33^ Regarding the formation of microstructures, melt-crystallization at low supercooling or at high temperatures leads to the development of large banded spherulites. They are grown from twisting lamellae,^17,33,35–37^ driven by the chirality of the polymer chain and/or uneven surface stresses of the lamellae.^37,38^ In contrast, increasing supercooling yields smaller spherulites due to an increase of the nuclei density and, simultaneously, a decrease in band spacing.^36,37^ The maximum growth rate of P3HB spherulites is about 4 μm/s at temperatures of 80–90 °C.

Despite these numerous investigations of the P3HB crystallization kinetics at low supercooling, there is a lack of information regarding the crystallization kinetics of P3HB near *T*_g_ or at high supercooling of the melt, particularly concerning homogeneous crystal nucleation. This knowledge is crucial for polymer manufacturing, as polymer solidification often occurs under high supercooling conditions or at relatively low temperatures, near ambient conditions. If crystallization occurs under these conditions, homogeneous crystal nucleation may play an important role in controlling structure formation and, eventually, properties. To our knowledge, only a single study has addressed this aspect using simple hot-stage microscopy. This involved counting the number of formed spherulites at specific temperatures, between 20 and 100 °C, as a function of time.^39^ However, in these experiments, the nucleation rate at temperatures near *T*_g_, between 20 and 40 °C, was obtained by cold crystallization of the quenched amorphous phase, implying that the obtained data might not accurately reflect the values corresponding to those specific nucleation temperatures.

Therefore, to fill this knowledge gap, the present work aims to investigate the kinetics of homogeneous crystal nucleation of P3HB using fast scanning chip calorimetry (FSC) and Tammann’s two-stage crystal nuclei development method. FSC with its high cooling and heating capacity, as well as short time constant,^40^ allows realization of well-defined thermal/nucleation pathways and can yield quantitative information about the kinetics of homogeneous crystal nucleation in a broad temperature range. Tammann’s approach is based on the experimental observation that the temperatures of maximum nucleation and growth rates often are largely different.^41^ Therefore, it is possible to first form nuclei in a low-temperature nucleation-step which only can grow to crystals of detectable size in a high-temperature, growth/development-step. With this method, the number of detected growing crystals at the development stage (high temperature) is assumed to be the same as the number of nuclei formed during the nucleation (low temperature). Using FSC, Tammann’s approach has been widely applied to various polymer materials such as poly­(l-lactic acid) (PLLA),^42,43^ poly­(butylene succinate) (PBS),^44^ poly­(butylene succinate-*ran*-butylene adipate) copolymer (PBSA),^45^ polyamides (PA),^46–48^ poly­(butylene isophthalate) (PBI),^49^ poly­(butylene terephthalate) (PBT),^50^ poly­(ethylene terephthalate) (PET),^51^ or poly­(ε-caprolactone) (PCL).^52,53^

Beyond the nucleation kinetics, a spike-modified Tammann’s method has been introduced to investigate the thermal stability of homogeneous crystal nuclei formed at specific temperatures and was successfully applied to few polymers such as PLLA,^43^ PBSA,^45^ and PBI.^54^ The approach is similar to the classical Tammann experiment, however, it includes a short excursion of the system to a predefined temperature (spike temperature) on the transfer of nuclei to the growth stage, with the spike temperature being higher than the growth-stage temperature. Basically, the nuclei formed in the nucleation step at a certain temperature exhibit a nucleation-time dependent size-distribution. By applying various spike temperatures, this method enables a selective dissolution of nuclei smaller than the critical size associated with the corresponding spike temperature, effectively filtering the size distribution. For PLLA, PBSA, and PBI, under the given nucleation conditions, the number of nuclei gradually decreases as the spike temperature increases, and all nuclei are destroyed upon heating them to 85–90 K above the temperature of their formation. However, due to the very limited number of available studies, it is not known yet whether this is a general rule.

Summarizing the scope of this study, we attempt to provide quantitative knowledge about (i) the kinetics of formation, (ii) the thermal stability, and (iii) the size distribution of homogeneous crystal nuclei in P3HB. For this, we apply and further test our earlier developed experimental strategies, in particular the recent modifications of the nuclei-transfer step in Tammann’s method. The results in this work provide the necessary quantitative knowledge for controlling the microstructure via the crystallization process under processing-relevant rapid cooling conditions, thereby enabling the possibility to tailor final polymer properties and improving manufacturing efficiency. As a prominent example, the often reported brittleness of P3HB may effectively be reduced by increasing the nuclei density, by decreasing the size of spherulites.^55^

## 2 Experimental Section

### 2.1 Material

We used P3HB with a molecular weight of approximately 500 kg/mol^19,56^ from Sigma-Aldrich Chemical Co., Inc., St. Louis, Missouri, USA), delivered as white powder.

### 2.2 Fast Scanning Chip Calorimetry (FSC)

Power compensation Flash DSC 1 and 2+ (Mettler-Toledo, Greifensee, Switzerland) connected to Huber TC100 intracoolers (Peter Huber Kältemaschinenbau SE, Offenburg, Germany), employing UFS-1 chip sensors with a circular heatable area of 500 μm in diameter,^57^ were used in the present work for analysis of the kinetics of homogeneous crystal nucleation and crystallization at high supercooling of the melt. The sensor compartment was purged with nitrogen gas at a flow rate of 40 mL/min and the sample-support temperature was set constant at −90 °C. The empty sensor was conditioned and temperature-corrected according to the instrument operating instructions prior to placing a sample onto the chip membrane. The as-received P3HB powder was used without any further treatment.

### 2.3 Differential Scanning Calorimetry (DSC)

A heat-flux DSC 1 (Mettler-Toledo, Greifensee, Switzerland) operated in combination with a Huber TC100 intracooler (Peter Huber Kältemaschinenbau SE, Offenburg, Germany) was used to obtain information about the crystallization kinetics at high temperatures or low supercooling of the melt. The furnace was purged with nitrogen gas at a flow rate of 60 mL/min, and the instrument calibration was checked by measuring the temperature and enthalpy of melting of an indium standard. As-received P3HB powder with a mass of about 4 mg was encapsulated in 20-μL aluminum pans.

## 3 Results and Discussion

### 3.1 Critical Cooling Rates for Suppression of Crystallization and Nucleation

Figure 1 shows the temperature–time protocol for analysis of non-isothermal crystallization of P3HB (a) and the normalized enthalpy of crystallization as a function of cooling rate (b). The measurements included cooling the equilibrium melt from *T*_m,0_ (195 °C)^17–19^ to below *T*_g_ (observed at about 5 °C during cooling the melt at 1000 K/s) at various rates between 10 and 1000 K/s. For lower cooling rates, the melt was first rapidly cooled at 1000 K/s to 140 °C to avoid thermal degradation of the samples, before further cooling at the rate of interest. Due to the low signal-to-noise ratio of the heat-flow rate on slow cooling, the fraction of crystals cannot be directly measured. Therefore, the effect of the cooling rate on the achieved crystallinity was analyzed from subsequently recorded heating scans, collected at 1000 K/s. The enthalpy of crystallization during cooling, which corresponds to the total enthalpy change in the heating scan, was normalized to its maximum value (obtained at 10^–1^ K/s) and then plotted as a function of cooling rate, as shown in Figure 1b. Squares and circles represent data obtained on different samples, providing information about the reproducibility. The black line was drawn to guide the eye. With increasing cooling rate higher than 10^–1^ K/s, the crystallinity decreases in a rather narrow range and becomes zero at around 2 K/s, being the critical cooling rate for suppressing crystallization (CR1) (see the black arrow). Though crystallization can be inhibited by cooling faster than 2 K/s, formation of crystal nuclei during cooling may still occur. Prior to analysis of isothermal crystallization at specific temperatures, the critical cooling rate for suppressing nuclei formation (CR2) was investigated, as illustrated in Figure 2.

**Figure 1:**
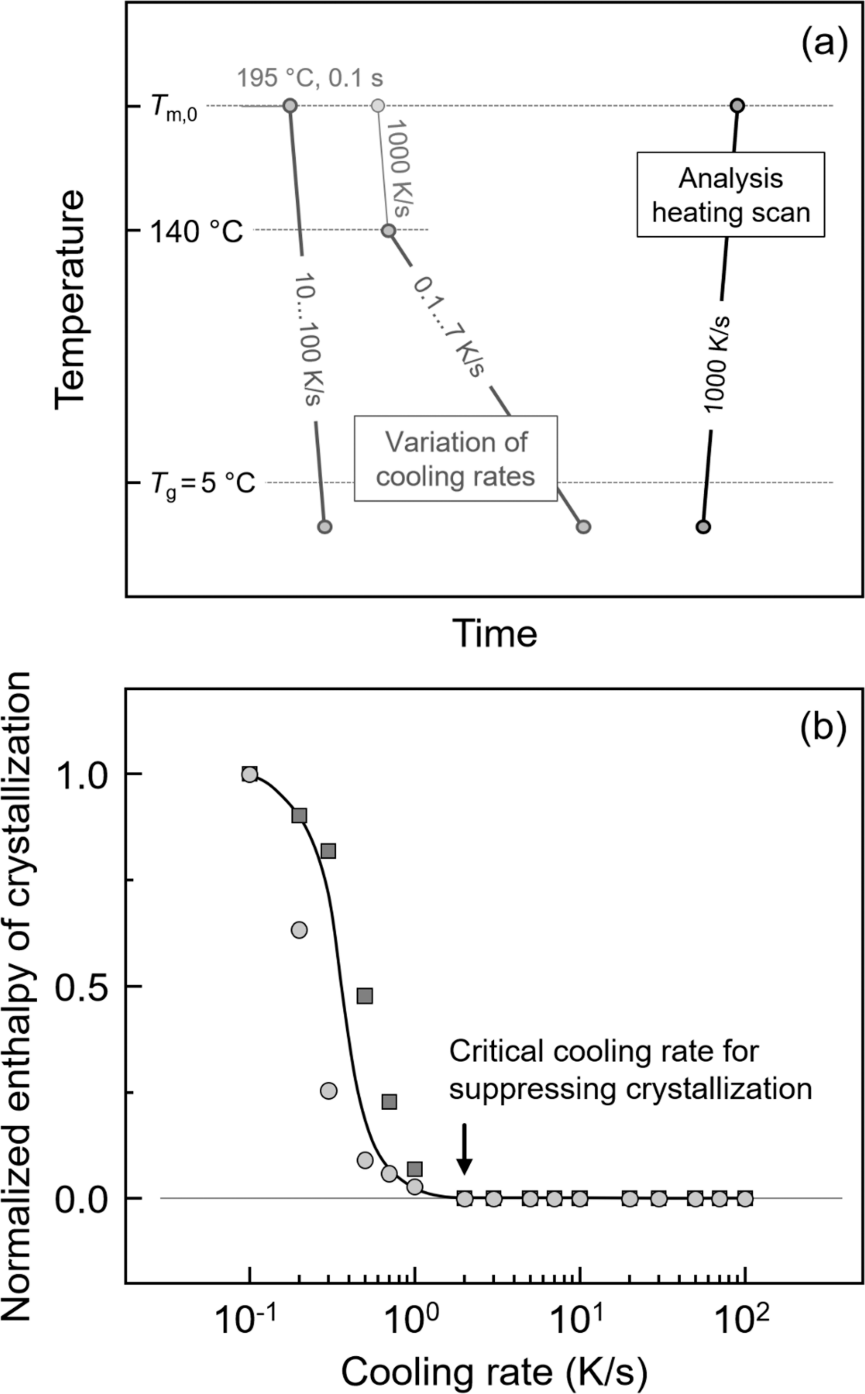
(a) Temperature–time protocol for analysis of the critical cooling rate for suppressing crystallization (CR1). (b) Normalized enthalpy of crystallization as a function of cooling rate. Different symbols in the bottom plot represent data obtained on two different samples, demonstrating reproducibility. The black line serves to guide the eye.

**Figure 2:**
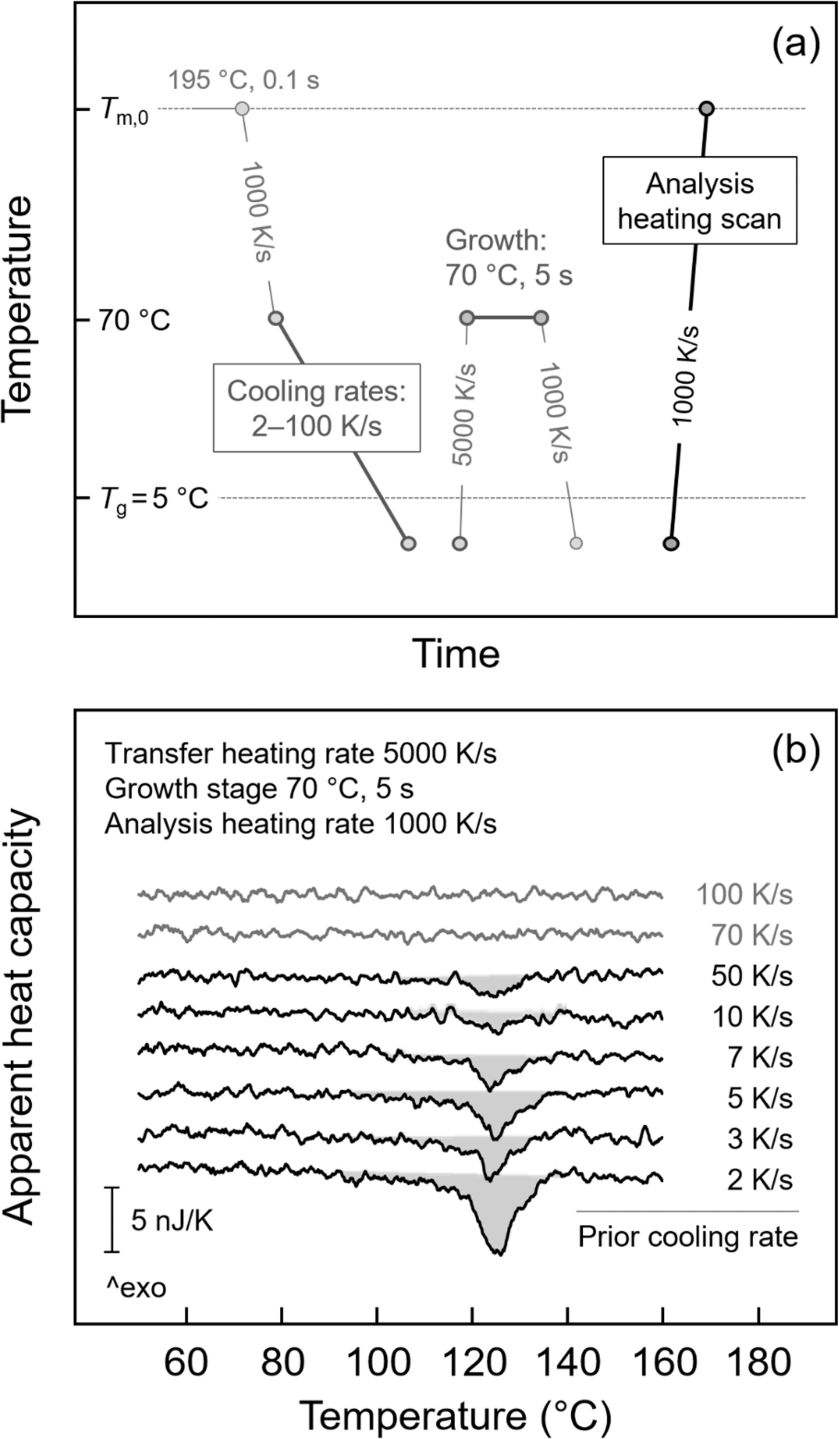
(a) Temperature–time protocol for analysis of the critical cooling rate for suppressing formation of nuclei (CR2). (b) Heating scans collected at 1000 K/s, after cooling P3HB with various rates to below *T*_g_, and transferring the system at 5000 K/s to the growth stage at 70 °C. The heating scans were corrected by subtracting a reference curve collected at 1000 K/s after prior cooling at 100 K/s.

Figure 2a presents the temperature–time protocol for analysis of the critical cooling rate to suppress formation of homogeneous crystal nuclei (CR2), employing Tammann’s two-stage crystal nuclei development method. The melt was first cooled at 1000 K/s to 70 °C and then cooled at various rates higher than the critical cooling rate CR1, required for preventing crystallization to 0 °C, that is, to slightly below *T*_g_. It is important to note that the effect of the cooling rate on the formation of nuclei was analyzed only in the temperature range below 70 °C, that is at temperatures lower than the temperature of the maximum crystallization rate, where homogeneous crystal nucleation is expected predominant.^33^ Then, the sample was immediately transferred at a rate of 5000 K/s, which does not allow nuclei formation during heating (as further outlined in more detail in Section 3.3), to the isothermal nuclei-development stage at 70 °C, to allow crystal growth for 5 s. If nuclei form during cooling and their sizes are supercritical at the growth stage, then these nuclei can grow to crystals, with their fraction analyzed on subsequent heating at 1000 K/s.

For demonstration, Figure 2b shows such analysis heating scans collected at 1000 K/s, with the data corrected by subtracting a reference curve. The reference curve was obtained after cooling the sample at 100 K/s, which suppressed crystallization and therefore melting in the analysis heating scan. As the cooling rate decreased to 50 K/s, a melting peak was observed, and its size increased with further lowering of the cooling rates, proving an increase in the number of supercritical nuclei. In summary, the experiment of Figure 2b suggests a critical cooling rate CR2 for suppressing formation of nuclei of approximately 70 K/s.

### 3.2 Kinetics of Isothermal Melt-Crystallization

Figure 3 illustrates the temperature–time protocol for analysis of the kinetics of crystallization at specific temperatures (a) and the characteristic times of crystallization as a function of temperature (b).

**Figure 3:**
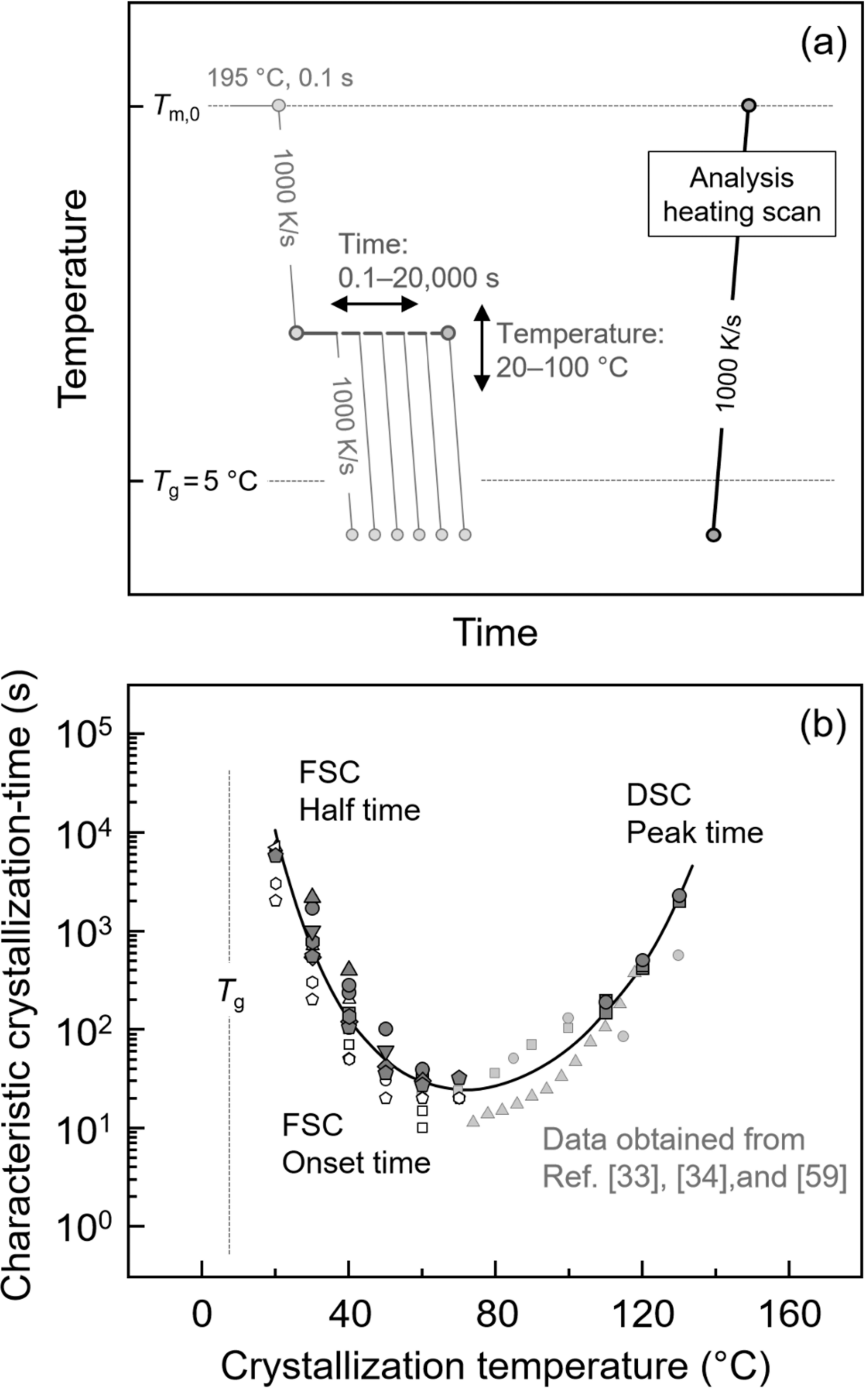
(a) Temperature–time protocol for analysis of the kinetics of crystallization at specific temperatures. (b) The characteristic time of crystallization as a function of temperature. Half- and peak-times of crystallization, presented by solid black symbols, were evaluated at high and low supercooling of the melt using FSC and DSC analyses, respectively. The onset time of crystallization obtained by FSC is presented by open black symbols. Various symbols represent data obtained on different samples, demonstrating reproducibility. Crystallization times obtained from ref 33, 34, and 59, represented in gray symbols, are inserted for comparison. Data from ref 33 and 34 are licensed under CC BY 4.0 and CC-BY-NC-ND 4.0, respectively. Data from ref 59 were reproduced with permission from Wiley.

To ensure absence of crystal and nuclei formation during cooling the melt to predefined crystallization temperatures, the relaxed melt was cooled at 1000 K/s to crystallization temperatures between 10 and 80 °C, with a 10 K increment. Due to the low signal-to-noise ratio of the heat-flow rate in FSC when crystallization stretches over long times, a direct determination of the crystallization kinetics is not possible. Instead, an indirect analysis approach was employed, involving the interruption of the isothermal crystallization process and determination of the crystallization-induced enthalpy change in the subsequently measured heating scan, as described in more detail elsewhere.^58^ Plotting the enthalpy change as a function of the crystallization time yields the crystallization half-time, defined as the time at which 50% conversion is achieved, as well as the onset time of crystallization, that is the time of first detection of an enthalpy of crystallization.

Besides FSC, DSC was employed to gain information about the crystallization at higher temperatures. In that case, the P3HB melt was linearly cooled at 60 K/min from 190 °C to predefined crystallization temperatures between 110 and 130 °C. In DSC experiments, the kinetics of crystallization was evaluated by the time of the maximum exothermic heat-flow rate, which is an empirical approach and provides a close approximation of the crystallization half-time.^34^

Figure 3b presents the characteristic time of crystallization of P3HB as a function of temperature. Different black symbols represent data obtained in this work from different samples, providing information about the reproducibility. Solid black symbols at high (below 80 °C) and low melt-supercooling indicate half- and peak-times of crystallization analyzed by FSC and DSC, respectively, and open symbols at low temperatures present the time at which crystallization begins, that is the onset time of crystallization. Gray symbols represent data available in the literature,^33,34,59^ for comparison. As such, P3HB exhibits a slightly asymmetric monomodal temperature-dependence of the crystallization time, with a minimum of about 30 s at 60–70 °C. This result is consistent with previously reported data (gray symbols).

### 3.3 Kinetics of Homogeneous Crystal Nucleation Using Tammann’s Two-Stage Crystal Nuclei Development Method

Prior to investigating the kinetics of homogeneous crystal nucleation, the critical nuclei-transfer heating rate for suppressing formation of additional nuclei and nuclei reorganization/stabilization during the transfer from the isothermal nucleation to the crystal-growth stages was analyzed, as shown in Figure 4. Figure 4a shows the temperature–time profile for such analysis. The P3HB melt was cooled at 1000 K/s to different nucleation temperatures near *T*_g_, followed by isothermal annealing for different times. It should be noted that the maximum time for nucleation at each temperature is limited by the time at which crystal growth starts (see open black symbols in Figure 3b). Then, the sample was transferred at various heating rates between 1 and 10,000 K/s (see the blue and red segments) to the growth stage, set at 70 °C, for permission of crystallization within 5 s, which is short enough to minimize any potential formation of additional nuclei at the growth stage (as shown by data in Figure 6c). The sample was then quenched to below *T*_g_, and subsequent heating scans at 1000 K/s were collected for evaluation of the crystal fraction formed during the growth stage via the enthalpy of crystallization.

**Figure 4:**
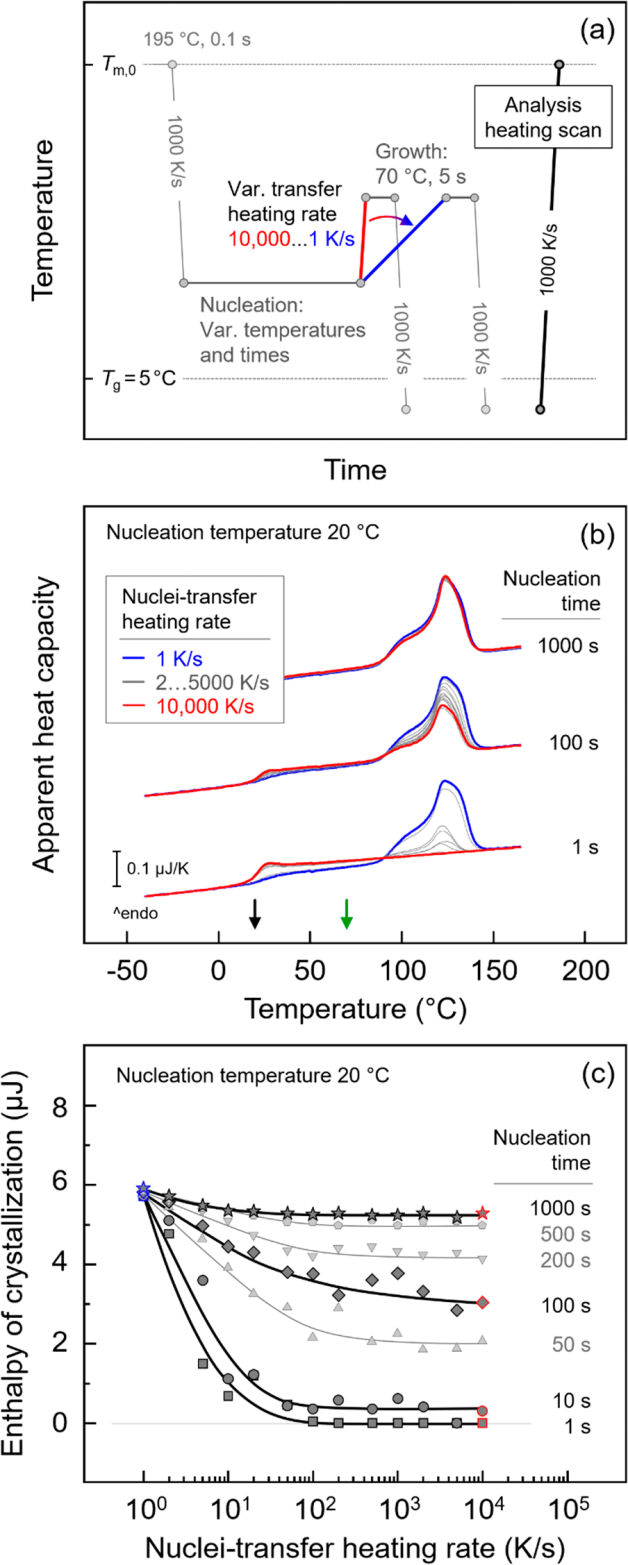
(a) Temperature–time protocol for analysis of the effect of the nuclei-transfer heating rate on the number of surviving nuclei, (b) sets of FSC heating scans collected at 1000 K/s, after transferring P3HB at different rates from the nucleation stage at 20 °C to the growth stage at 70 °C, and (c) enthalpy of crystallization as a function of the nuclei-transfer heating rate. The blue and red segments in (a) signify the lowest and highest transfer-heating rate, respectively, and correspond to the blue and red data shown in (b) and (c). Black and green arrows in (b) indicate the nucleation and growth temperatures, respectively.

Figure 4b shows exemplary sets of heating curves collected at 1000 K/s after transferring the sample, which was nucleated at 20 °C for various time, with different rates to the growth stage at 70 °C, as described in the thermal protocol shown in Figure 4a. The heating scans associated with the lowest and highest transfer-heating rate are presented in blue and red colors, respectively, while curves associated with in-between rates are plotted gray. The black and green arrows at the temperature axis indicate the nucleation and growth temperatures, respectively. When the nucleation time is fixed at 1 s (bottom set of curves) and a transfer-heating rate of 1 K/s is used (blue curve), multiple melting events in the temperature range between 80 and 150 °C are detected. The lowest melting peak, appearing as a shoulder at around 100 °C, is associated with the melting of crystals formed either during the nuclei transfer or at the growth stage, while the melting peaks higher than that are related to the melting of reorganized crystals; note that we are not aware of studies about the crystal-reorganization kinetics, which would have provided a critical heating rate to suppress crystal reorganization. With increasing transfer heating rate, the melting peaks decrease rapidly in size (see gray curves) and disappear if the heating rate is higher than 100 K/s. The decrease of the melting-peak area/melting enthalpy is caused by a reduction of the number of supercritical-size nuclei present in the sample at the beginning of the growth stage and indicates suppression of stabilization/growth or formation of such nuclei during the nuclei transfer stage.^53^ Finally, the zero enthalpy of crystallization indicates the complete absence of supercritical nuclei at the growth stage. At high transfer heating rates, such nuclei must be formed at the nucleation stage since, contrary to lower transfer heating rates, there is no possibility for growth of smaller nuclei to the required size during the transfer.

With the longer nucleation time of 100 s (center set of curves), the melting-peak area similarly decreases with increasing transfer heating rate up to 1000 K/s. However, it becomes steady and remains non-zero even on heating at 10,000 K/s. Obviously, nucleation at 20 °C for 100 s produces broadly distributed sub- and supercritical-size nuclei, with subcritical-size nuclei not surviving the transfer to the growth stage if the transfer-heating rate is higher than about 1000 K/s. Only nuclei with a size supercritical at both the nucleation and growth stages ultimately contribute to crystallization.

At the maximum nucleation time of 1000 s realized in this experiment (top set of curves), the melting-peak area is independent of the transfer-heating rate. This observation suggests that nucleation for 1000 s results in the formation of a large number of supercritical (at the growth stage temperature) homogeneous crystal nuclei. At the development stage, these nuclei develop a space-filling morphology, and even an increase in the number of supercritical nuclei at the lowest transfer heating rates does not significantly alter the crystallization enthalpy.

For better visualization of the data, Figure 4c provides quantitative information about the effect of the nuclei-transfer heating rate on the relative number of homogeneous crystal nuclei at the development stage, as measured by the enthalpy of crystallization. Blue and red symbols represent the data obtained at the transfer heating rates of 1 and 10,000 K/s, respectively, and correspond to the coloring of the heating curves in Figure 4b. At the lowest transfer-heating rate of 1 K/s, the crystallization enthalpies of the sample obtained at various nucleation times are identical, indicating the creation of a similar large number of supercritical nuclei during the slow transfer. With increasing transfer-heating rate from 1 to 100 K/s, regardless of the nucleation time, the crystallization enthalpy decreases and reaches a plateau at a level that depends on the nucleation time. The longer the nucleation time, the higher is the final (plateau) crystallization enthalpy/nuclei number. Data are interpreted such that nuclei forming in the nucleation step exhibit a size distribution, with a large number of nuclei of supercritical size at the nucleation temperature of 20 °C. If the transfer-heating rate is low, further stabilization of the formed nuclei or even formation of additional nuclei during heating may occur, eventually affecting the number of supercritical nuclei present at the growth stage temperature. With increasing the transfer-heating rate up to 100 K/s, both the stabilization of existing nuclei and the formation of additional nuclei are gradually suppressed, causing a decrease in the number of supercritical nuclei and, consequently, in the crystallization enthalpy. As a further increase of the transfer-heating rate does not cause a further decrease of the nuclei number/crystallization enthalpy, this heating rate is defined as the critical transfer-heating rate above which nuclei stabilization and formation cannot occur, at least for the selected combination of nucleation and growth temperatures. Finally, the different plateau levels of the crystallization enthalpy, obtained when using a supercritical transfer heating rate higher than 100 K/s, indicate the nucleation-time dependence of the number of nuclei at 20 °C being of supercritical size at the growth-stage temperature of 70 °C.

In a recent study of homogeneous crystal nucleation in PBSA, a nucleation- and growth-stage-temperature dependent transfer-heating rate was suggested, considering that a larger temperature difference between the nucleation and the growth stages results in a longer nuclei-transfer time.^45^ In order to accurately investigate the rate of homogeneous crystal nucleation at specific temperatures, it is therefore essential to define an appropriate nuclei-transfer heating rate as otherwise the number of supercritical nuclei detected at the growth stage and the number evident in the nucleation stage may differ. As such, Figure 5 illustrates the effect of the nucleation temperature, including −10 (a), 0 (b), 10 (c), and 30 °C (d), on the critical transfer-heating rate.

**Figure 5:**
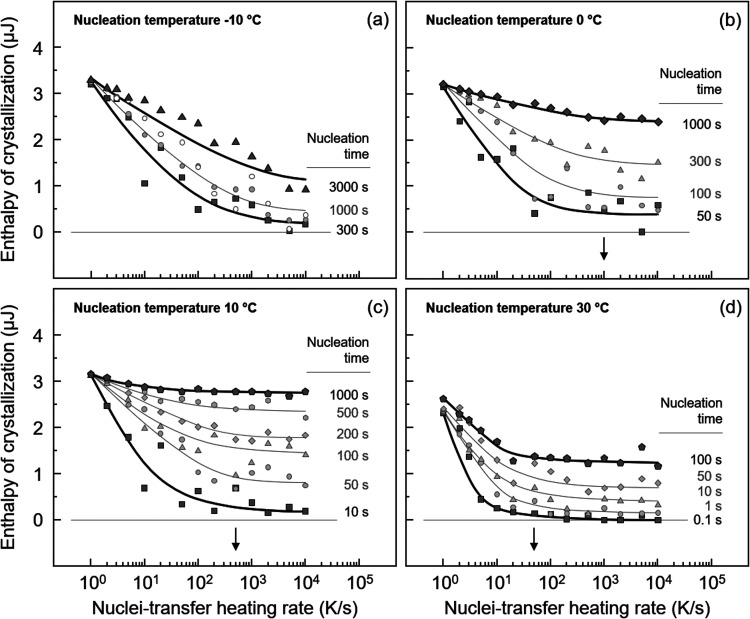
Enthalpy of crystallization at the growth stage as a function of the nuclei-transfer heating rate of P3HB, nucleated at −10 (a), 0 (b), 10 (c), and 30 °C (d) for different times. The growth conditions are identical in all experiments, being at 70 °C for 5 s. Black arrows indicate the approximate critical transfer-heating rate altered by applying different nucleation temperatures.

When compared to the data obtained at the nucleation temperature of 20 °C (Figure 4c), if a higher nucleation temperature of 30 °C is used (Figure 5d), the critical transfer-heating rate is reduced to about a few ten K/s (see black arrow). In contrast, when nucleation temperatures below 20 °C are applied, the critical transfer-heating rate increases significantly, raising to about 500 and 1000 K/s for nucleation temperatures of 10 and 0 °C, respectively (as indicated by the black arrows). In the case of nucleation at −10 °C, the crystallization enthalpy continuously decreases as the transfer-heating rate increases within the analyzed heating-rate range. Even employing a heating rate of 10,000 K/s, which is the limit of the used instrument, the crystallization enthalpy does not level off, suggesting that the maximum rate of 10,000 K/s is still below the critical value required to inhibit growth/stabilization of nuclei during the transfer process. For this reason, the rate of homogeneous crystal nucleation was investigated only in the temperature range between 0 and 50 °C, using 1000 K/s as the transfer-heating rate.

The temperature dependence of the homogeneous crystal-nucleation rate was analyzed using Tammann’s two-stage crystal nuclei development method, as shown in Figure 6. Figure 6a presents the temperature–time protocol for this analysis. Figure 6b shows the enthalpy of crystallization as a function of nucleation time for selected temperatures, with data obtained from the analysis heating scans. Finally, Figure 6c illustrates the characteristic time of nucleation (blue symbols) and crystallization (black) of P3HB as a function of temperature. The different symbols in Figure 6c represent data collected from different samples, indicating the reproducibility of the obtained data.

**Figure 6:**
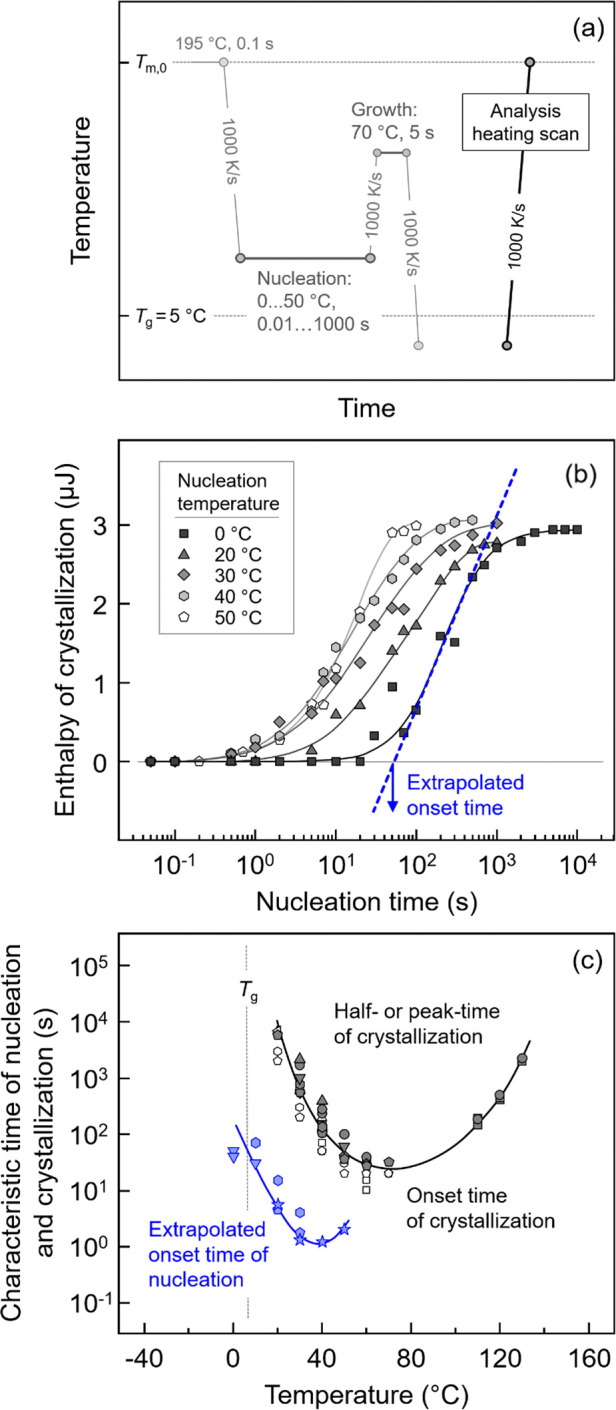
(a) Temperature–time protocol for analysis of the kinetics of homogeneous crystal nucleation using Tammann’s two-stage crystal nuclei development method. (b) Enthalpy of crystallization as a function of nucleation time for selected temperatures, with data derived from the analysis heating scan. The blue arrow and dashed line demonstrate the way to estimate the onset time of homogeneous nucleation. (c) Characteristic time of nucleation (blue symbols) and crystallization (black) of P3HB as a function of temperature. Various symbols represent data obtained on different samples to confirm reproducibility.

Regarding Figure 6a, the relaxed melt was cooled at 1000 K/s to the nucleation temperature between 0 and 50 °C and then annealed for different times. The maximum nucleation time at each temperature was defined by the onset time of crystallization (see open black symbols in Figures 3b or 6c). Next, the sample was transferred at 1000 K/s to the growth stage set at 70 °C for 5 s and then recooled to below *T*_g_, before recording the analysis-heating scan at 1000 K/s. If the nucleation time is too short, then there is no formation of supercritical nuclei, which could grow into crystals at 70 °C within 5 s. However, when the sample is annealed for a sufficiently long time, nuclei, supercritical in size even at the growth temperature, form in the nucleation stage, and those nuclei grow into crystals, with their fraction analyzed by the enthalpy of crystallization in the analysis heating scan. With increasing nucleation time, the number of such supercritical nuclei increases, leading to a corresponding increase of the crystallization enthalpy, as illustrated in Figure 6b. Due to data fluctuations at the initial stage of nucleation, the extrapolated onset time is determined instead of the time of the first observation of non-zero crystallization enthalpy. The onset time of homogeneous crystal nucleation was estimated by extrapolating the steepest slope of the crystallization-enthalpy curve to the zero-enthalpy baseline, as shown by the blue arrow and dashed line. The obtained values were then plotted as a function of temperature, as illustrated by the blue symbols in Figure 6c. The characteristic times of crystallization are also added to the plot for comparison (black symbols). The data reveal that homogeneous nucleation begins at a time about two orders of magnitude shorter than crystallization, with the shortest nucleation onset-time of 1 s detected at 30–40 °C, that is, about 30 K above *T*_g_.

### 3.4 Thermal Stability of Homogeneous Crystal Nuclei Using a Spike-Modified Tammann’s Two-Stage Crystal Nuclei Development Method

In this part, the thermal stability of homogeneous crystal nuclei of P3HB, formed at specific temperatures near *T*_g_, is investigated employing a spike-modified Tammann’s two-stage crystal nuclei development method, as presented in Figure 7. Figure 7a shows the temperature–time protocol for analysis of the thermal stability of crystal nuclei. The quiescent melt was cooled at 1000 K/s to the nucleation temperature between 0 and 30 °C and then annealed for different times. During crystal nucleation at a given temperature, nuclei with a nucleation-time dependent size distribution form. To selectively analyze the size distribution, varying spike temperatures were employed during the transfer of the nuclei to the growth stage at 1000 K/s. Different spike temperatures, ranging from 75 to 150 °C (with an interval of 5 K), were used, while the spike time was kept constant at 0.01 s. This short temperature excursion before the growth stage facilitates the dissolution of nuclei that are smaller than the critical size at each spike temperature, leaving only those nuclei that are larger. By increasing the spike temperature, the size distribution of the supercritical nuclei is successively truncated at the small side. The remaining nuclei, all above the supercritical size for the growth temperature, then undergo subsequent growth at 70 °C. After the growth stage, the crystallinity developed within 5 s was analyzed by the final analysis heating scan at 1000 K/s.

**Figure 7:**
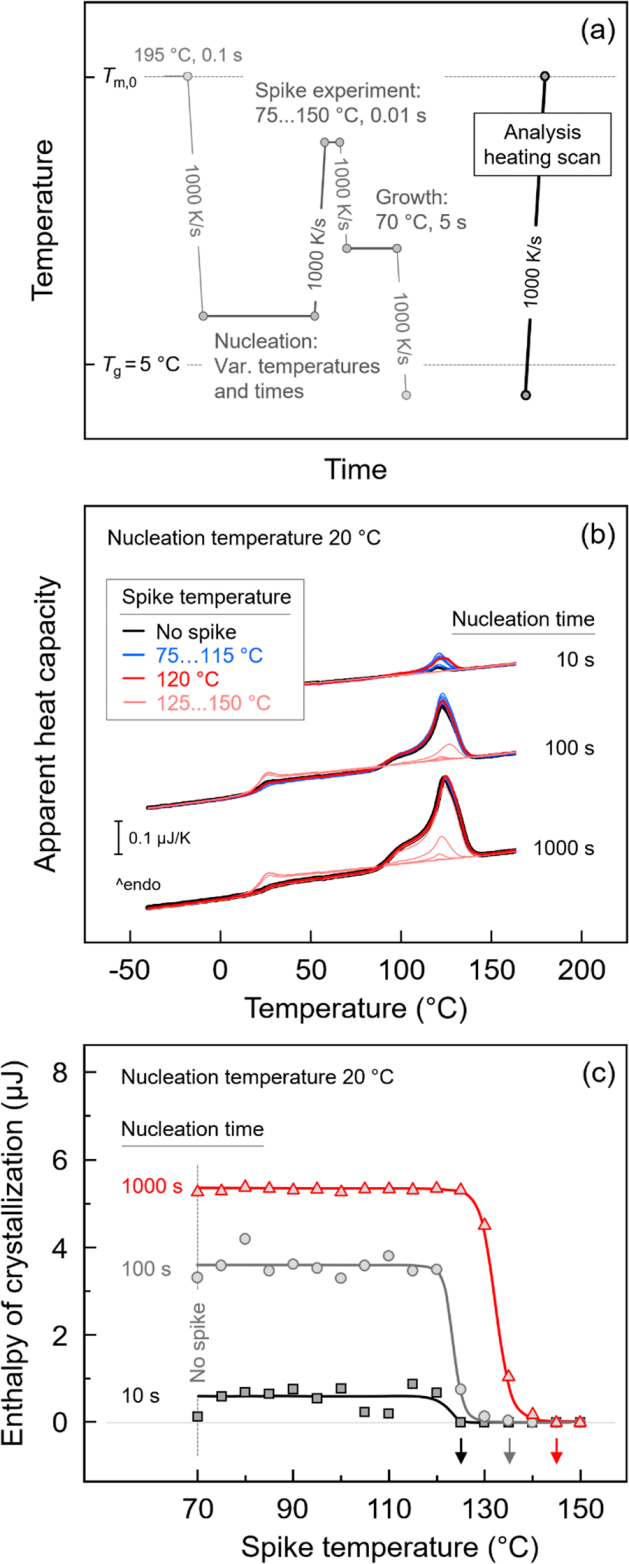
(a) Temperature–time protocol for analysis of the thermal stability of homogeneous crystal nuclei of P3HB using a temperature-spike-modified Tammann’s method, (b) exemplary sets of heating scans collected at 1000 K/s, obtained after transferring P3HB nucleated at 20 °C for 10, 100, and 1000 s as indicated to different spike temperatures at 1000 K/s and subjecting the system to growth at 70 °C for 5 s, and (c) enthalpy of crystallization of P3HB at the growth stage as a function of spike temperature. The vertical gray line indicates the growth temperature and absence of a spike.

For demonstration, Figure 7b presents exemplary sets of final heating scans of P3HB, which was nucleated at 20 °C for 10 (top set of curves), 100 (center), and 1000 s (bottom). The differently colored curves correspond to different spike temperatures, as indicated in the legend. When the nucleation time is set to 1000 s (bottom set of curves), but sufficiently short to avoid crystallization (see black open symbols in Figures 3b or 6c), and without applying the spike (black curve), a large melting peak is detected. When applying a spike up to a temperature of about 120 °C (red curve), the size of the melting peaks remains constant. However, with a further increase of the spike temperature, the melting peak decreases in size (light-red curves) and becomes zero when the spike temperature is above 140 °C. When shorter nucleation times and no spike are applied (black curves of center and top curve sets), only tiny melting peaks are observed, suggesting a much lower number of supercritical nuclei, as expected. When applying temperature spikes, these two sets of curves exhibit a similar behavior as the data obtained with the nucleation time of 1000 s: the size of the melting peaks remains relatively constant up to a certain temperature before a drastic decrease down to zero.

To better demonstrate these behaviors, Figure 7c shows the crystallization enthalpy as a function of the spike temperature, with the vertical gray line indicating the growth temperature. Regardless of the nucleation time, the crystallization enthalpy remains constant up to a certain spike temperature before decreasing to zero in a narrow temperature range, indicating complete dissolution of all nuclei. The drastic drop of the crystallization enthalpy suggests that after annealing at 20 °C only few nuclei are stable above about 120 °C. With increasing nucleation time the rapid drop in crystallization enthalpy/number of growing nuclei shifts to slightly higher temperatures, e.g., 130 °C after 1000 s. In other words, homogeneous nuclei of P3HB formed at the given condition show a distribution with a rather sharp cutoff at large sizes. With the increase of the nucleation time, the stability of the nuclei slightly increases, as evidenced also by a horizontal shift of the final dissolution temperature to higher values (see colored arrows at the temperature axis).

In order to confirm the rapid drop of the nucleus-size distribution for larger nuclei and the relatively high stability of the nuclei, we performed similar experiments also at other nucleation temperatures. Figure 8 shows the enthalpy of crystallization of P3HB at 70 °C for 5 s as a function of the spike temperature, obtained after nucleation at 0 °C (a), 10 °C (b), and 30 °C (c) for different times. As before in Figure 7c, the vertical gray line in each plot indicates the growth temperature. For all nucleation temperatures of 0, 10, and 30 °C, regardless of the nucleation time, the crystallization enthalpy exhibits a similar behavior as in case of nucleation at 20 °C, shown in Figure 7c. At first, the number of supercritical nuclei remains almost constant, or decreases only slightly when the spike temperature is below 120 °C, and then drops suddenly when the spike temperature is above 120 °C.

**Figure 8:**
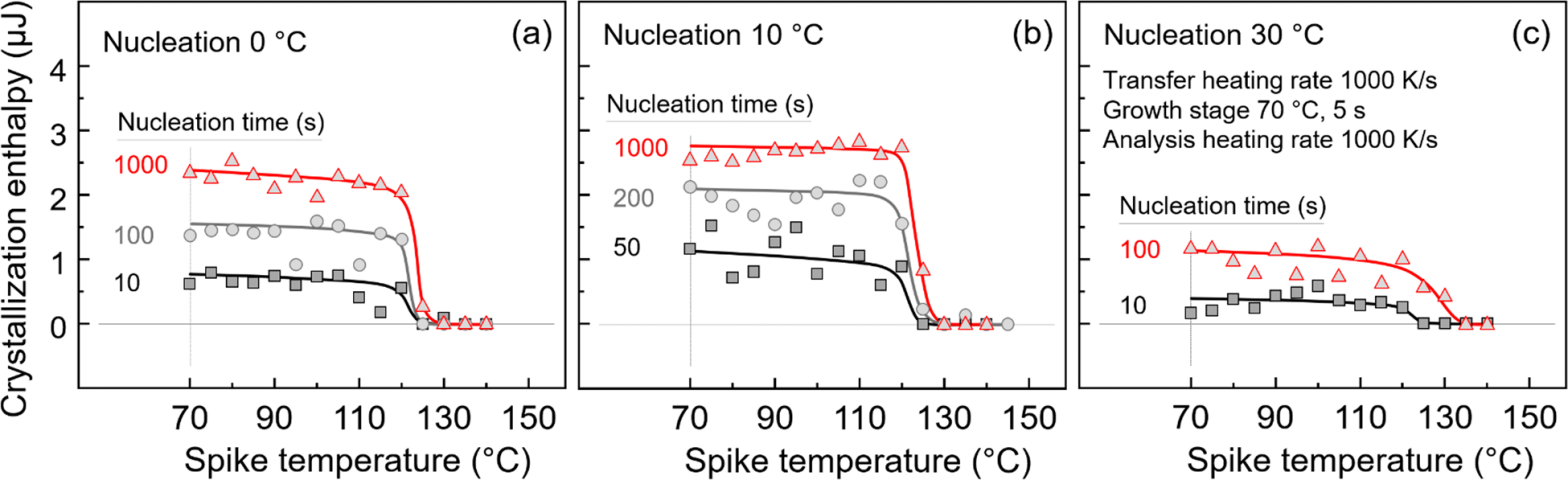
Enthalpy of crystallization of P3HB developed during the growth stage as a function of the spike temperature. Data were obtained for different nucleation temperatures: (a) 0 °C, (b) 10 °C, and (c) 30 °C, each for different times. The transfer-heating rate was 1000 K/s, and the growth temperature was set 70 °C, lasting for 5 s. The vertical gray lines indicate the growth temperature and data obtained without exposing the system to an additional temperature spike.

In summary, at the given nucleation conditions near *T*_g_, the number of supercritical nuclei remains constant in a large spike-temperature interval and drastically decreases within 10–15 K if the spike temperature exceeds 120 °C. In other words, nuclei are only destroyed about 90 to 120 K above their formation temperature. When compared to other polymers, such as PLLA, PBSA, and PBI, the number of nuclei in these materials gradually decreases as the spike temperature increases, and all nuclei are destroyed around 85–90 K above the temperature of their formation. The “melting” interval for these three polymers is approximately 25 K for PLLA, 30 K for PBSA, and 40 K for PBI. As such, the narrower “melting” interval of P3HB nuclei of <15 K suggests a much sharp cutoff of the nuclei-size distribution at large size.

Based on classical nucleation theory (CNT),^60,61^ the temperature-dependence of the critical radius of clusters (*R*_c_) can be estimated using eqs 1 and 2:^43,62^

$$R_{c}\left(T\right)=\frac{2\sigma }{\Delta g\left(T\right)}$$ 1

$$\Delta g\left(T\right)=\Delta h_{m}\left(\frac{T_{m,0}-T}{T_{m,0}}\right)\left[1-\frac{\Delta c_{p}\left(T_{m,0}\right)}{\Delta s_{m}\left(T_{m,0}\right)}\frac{\left(T_{m,0}-T\right)}{2T_{m,0}}\right]$$ 2

Here, σ is the interfacial free energy between the liquid and crystal phases, Δ*g* is the thermodynamic driving force for the crystallization process, Δ*h*_m_ and Δ*s*_m_ are the bulk enthalpy and entropy of melting at the equilibrium melting temperature *T*_m,0_, respectively, and Δ*c*_p_(*T*_m,0_) is the difference between the heat capacities of the liquid and crystal/solid phases at *T*_m,0_. Values of these parameters, used for calculation of *R*_c_(*T*), are provided in Table 1, in units reported in the original references. For converting mol-specific to volume-specific data, the available molar volume is used.

**Table 1:** Parameters Applied for Calculation of the Critical Radius of Clusters Using eqs 1 and 2 ^a^

parameter	value	ref
equilibrium melting temperature (*T*_m,0_)	468 K	^17,64,65^
interfacial free energy between liquid and solid phases (σ)	0.044 J/m^2^	^17,66,67^
specific bulk enthalpy of melting (Δ*h*_m_)	12,570 J/mol	^63^
	1.85 × 10^8^ J/m^3^	^17^
specific bulk entropy of melting (Δ*s*_m_ = Δ*h*_m_/*T*_m,0_)	26.859 J/K/mol	
specific heat capacity of solid PHB (*c*_p,solid_)	1.858 J/g/K	^68^
specific heat capacity of liquid PHB (*c*_p,liquid_)	2.073 J/g/K	^68^
molar volume (*V*_P3HB_)	7.5 × 10^–5^ m^3^/mol	^64^

a The molar volume is used for interconversion of units. All values hold for *T* = *T*_m,0_.

Figure 9a presents the estimated radius of critical-size clusters (*R*_c_) as a function of temperature. The red and blue curves refer to data calculated using different bulk enthalpies of melting reported in the literature,^17,63^ respectively, suggesting an only minor effect (see gray-shaded area between the curves). The critical cluster radius represents the size above which nuclei will preferably not dissolve at the temperature of formation but may grow with a reduction of their free enthalpy. In contrast, clusters smaller than this size will preferably dissolve. The plot shows that this critical size increases with temperature. As such, the minimum size/radius of supercritical nuclei is about 1.5 nm at room temperature while it asymptotically approaches infinity at *T*_m,0_. The link to the spike-experiments of Figures 7 and 8 is then given with Figure 9b, showing, as an example, the enthalpy of crystallization as a function of the spike temperature after nucleation at 20 °C for different times. The upper axis reports the minimum size of the clusters that are supercritical at the corresponding spike temperature, based on the data of Figure 9a. The vertical dashed lines in the plot indicate the maximum radius of the largest detected clusters, determined as intersection of the tangent at the steepest slope of the crystallization-enthalpy curve with the zero enthalpy-of-crystallization line.

**Figure 9:**
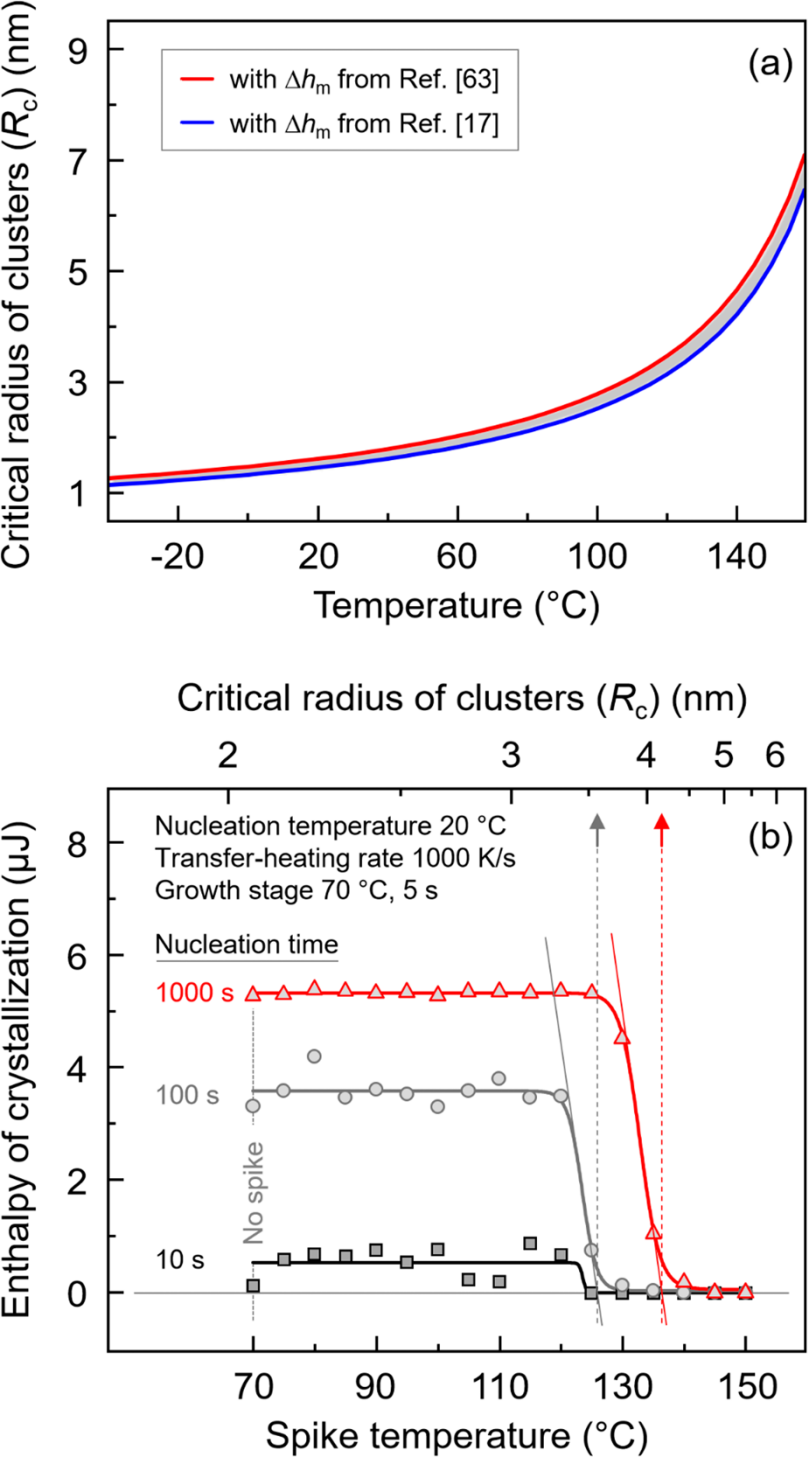
(a) Radius of critical-size clusters (*R*_c_) as a function of temperature, obtained by eqs 1 and 2 with values of the variables listed in Table 1. The red and blue curves illustrate data calculated using different values of the bulk enthalpy of melting reported in the literature.^17,63^ The gray-shaded area between the curves suggest a negligible effect. (b) An exemplary plot of the enthalpy of crystallization [=nuclei-number equivalent] as a function of the spike temperature at selected homogeneous nucleation conditions, as indicated. The critical size of nuclei that are stable at the corresponding spike temperature is indicated at the upper axis. Gray- and red-colored dashed lines and arrows indicate the size of the largest nuclei obtained on nucleation P3HB at 20 °C.

Regarding the longest nucleation time of 1000 s (red symbols), the enthalpy of crystallization, which is proportional to the number of surviving supercritical nuclei at the growth stage, decreases rapidly at spike temperatures between about 130 and 140 °C. The tangent construction (red dashed line) suggests a maximum cluster radius of roughly 4 nm (see red arrow). In case of shorter nucleation times, the radius of the largest clusters slightly decreases to about 3.5 nm (see gray arrow). Due to their smaller size, these clusters exhibit lower stability and are, therefore, dissolving at a slightly lower temperature. Though details of the structure (bulk and surfaces) as well as the shape of detected homogeneous P3HB crystal nuclei are unknown, the sharp truncation of the stability/size distribution may point to a limitation of one of the parameters controlling their thermodynamic stability, including their lateral size.

## 4 Conclusions

The kinetics of homogeneous crystal nucleation and stability of nuclei of poly­(3-hydroxybutyrate) (P3HB) were analyzed using fast scanning calorimetry (FSC) combined with Tammann’s two-stage crystal nuclei development method. Regarding non-isothermal ordering processes, crystallization of P3HB is suppressed if the melt is quenched to below the glass transition temperature (*T*_g_) at rates higher than a few K/s, while cooling at significantly higher rate of approximately 100 K/s is required to prevent the formation of nuclei.

Isothermal crystallization experiments revealed a monomodal temperature-dependence of the crystallization time of P3HB, with a minimum crystallization half-time of about 30 s at 60–70 °C. Homogeneous crystal nucleation is fastest at around 40 °C (30 K higher than *T*_g_), with an onset time of about 1 s, which is about two orders of magnitude shorter than the crystallization onset-time at the same temperature.

The thermal stability of nuclei was assessed employing a spike-modified Tammann protocol, applying a supercritical nuclei-transfer heating rate, necessary to probe the nuclei-size distribution at the nucleation temperature. The number of nuclei, formed near *T*_g_ with a supercritical size at the growth temperature, remains nearly constant upon heating to about 100 K above their formation temperature, before a rapid decrease on further heating. Complete dissolution of all nuclei was observed at about 100 to 120 K above the temperature of their formation. This result suggests that the distribution of the size of nuclei significantly stretches to much higher values (around 4 nm) than needed to survive at the nucleation temperature (around 1.5 nm at ambient temperature). This observation is in line with earlier nucleation studies on, e.g., poly­(l-lactic acid) (PLLA),^43^ poly­(butylene succinate-*ran*-butylene adipate) (PBSA),^45^ poly­(butylene isophthalate) (PBI),^54^ poly­(ethylene terephthalate) (PET),^51^ or even the low molecular mass compound tolbutamide,^69^ which all revealed fractions of low-temperature-formed nuclei, surviving temperature-jumps up to close 100 K above the temperature of formation, thus being much larger than the critical size at the nucleation temperature. However, in contrast to PLLA, PBI, or PBSA, the size distribution of the nuclei in PH3B seems much sharper truncated for large nuclei, as the last remaining nuclei dissolve in a very narrow temperature range well above the formation-temperature.

Considering that crystals typically melt only a few K above the crystallization temperature,^44,70,71^ the exceptional stability of fractions of the nuclei population forming at a specific temperature suggests a fundamentally different structure and growth mechanism than for crystals, needed to be explored in future works. The relatively large (calculated) size of the most stable nuclei of the order of magnitude of a few nm is not directly detectable by imaging or calorimetry (change of heat capacity, enthalpy of nuclei formation), which suggests that the size may be overestimated and that the calculation scheme, e.g. when assuming spherical nuclei, or the parameters (e.g., σ, Δ*h*_m_, Δ*s*_m_) to estimate critical nuclei sizes within the frame of the classical nucleation theory (CNT) may require modification.
